# 

**Published:** 1920-03

**Authors:** 


A JOURNAL OF THE MEDICAL SCIENCES FOR THE WEST OF ENGLAND AND SOUTH WALES.

*PUBLISHED UNDER THE AUSPICES OF*

*THE BRISTOL MEDICO-CHIRURGICAL SOCIETY.*

EDITOR:
P. WATSON-WILLIAMS, M.D.

WITH WHOM ARE ASSOCIATED

JAMES SWAIN, C.B., C.B.E., M.S., M.D., J. LACY FIRTH, M.S., M.D.,
CAREY COOMBS, M.D.

J. A. NIXON, C.M.G., M.D., Assistant Editor.

EDITORIAL SECRETARY:
J. M. FORTESCUE-BRICKDALE, M.A., M.D.

---

"Scire est nescire, nisi id me

Scire alius sciret."

*VOL. XXXVII.*

BRISTOL: J. W. ARROWSMITH LTD.
London: Simpkin, Marshall, Hamilton, Kent & Co. Limited.
1920.

J. W. Arrowsmith Ltd. Publishers Bristol

### CONTENTS OF VOLUME XXXVII.

*Page*

Queene Elizabethes Achademy. By L. M. Griffiths, M.R.C.S. Eng., L.R.C.P. Ed. .. .. .. .. .. .. .. .. 1

Some Considerations on the Applications of Physiology to Medicine. By G. A. Buckmaster, M.A., M.D. Oxon. .. .. .. 7

The Modern Treatment of Tuberculosis of the Spine. By A. Rendle Short, M.D., B.S., B.Sc., F.R.C.S. .. .. .. .. 19

On Cases of Encephalitis Lethargica in Bristol. By D. S. Davies, J. O. Symes, F. H. Edgeworth, I. Walker Hall and J. A. Nixon .. .. .. .. .. .. .. .. .. 25

Malaria. A Discussion by D. S. Davies, S. Kerfoot and Cecil Clarke .. .. .. .. .. .. .. .. 38

Case of Lymphosarcoma of the Tonsil and Palate. By P. Watson-Williams, M.D. Lond. .. .. .. .. .. .. 51

An Operation for the Cure of Prolapse and Cystocele. By Walter C. Swayne, M.D., B.S. .. .. .. .. .. .. 81

On the Causes of Death in Lobar Pneumonia. By F. H. Edgeworth, M.A., M.D., D.Sc. .. .. .. .. .. .. .. 88

A Case of Erythræmia or Splenic Polycythæmia. By G. Parker, M.A., M.D. Cantab. .. .. .. .. .. .. .. 91

The Effects of the War on the Mental Condition of the Citizens of Bristol. By R. H. Norgate, M.R.C.S., L.R.C.P. .. .. 95

Irritable Heart. A Discussion by Dr. C. F. Coombs, Dr. C. E. K. Herapath, and Dr. A. G. Morris .. .. .. .. .. 108

Famine-Dropsy as a Food-Deficiency Disease. By J. A. Nixon, C.M.G., M.D., F.R.C.P. .. .. .. .. .. .. 137

Vesical Calculus in a Urinary Typhoid Carrier. By Duncan Wood, F.R.C.S. .. .. .. .. .. .. .. .. 148

R31 B8

*Page*

Poisoning by "Mustard" Gas. By E. H. E. Stack, F.R.C.S., Capt., R.A.M.C., (T.F.), Carey F. Coombs, M.D., F.R.C.P., Capt., R.A.M.C. (T.F.), and R. Rolfe, B.A., M.B., B.Ch. Cantab. 151

Medicine and Surgery. By L. E. V. Every-Clayton, M.D. Lond. 193

The Diagnosis of Torsion of the Great Omentum, illustrated by two Case-Histories. By James Swain, C.B., M.S., M.D. Lond., F.R.C.S. .. .. .. .. .. .. .. .. 202

The Clinical Significance of Adsorption Phenomena. By Oliver C. M. Davis, M.D. Bristol .. .. .. .. .. .. 205

Tuberculosis and Consumption in Relation to Public Health. By D. S. Davies, M.D. Lond., LL.D., D.P.H... .. .. .. 211

Progress of the Medical Sciences:

Medicine .. .. .. .. .. .. 52, 124, 162

Surgery .. .. .. .. .. .. .. 55, 165

Reviews of Books .. .. .. .. 59, 127, 170, 224

Editorial Notes .. .. .. .. .. 73, 130, 178, 229

The Library of the Bristol Medico-Chirurgical Society 76, 133, 182, 234

Meetings of Societies .. .. .. .. .. 78, 135, 236

Obituary .. .. .. .. .. .. .. .. .. 184

Local Medical Notes .. .. .. .. 79, 136, 187, 242

### The Library of the Bristol Medico-Chirurgical Society.

*The following donations have been received since the publication of the list in December,* 1919.

*10th March,* 1920.

| | |
|---|---|
| C. W. J. Brasher, M.D. (1) .. .. .. | 29 volumes. |
| L. M. Griffiths (2) .. .. .. .. .. | 6 volumes. |
| Medical Research Committee (3) .. .. .. | 9 volumes. |

Unbound periodicals have been presented by Drs. J. M. Fortescue-Brickdale, J. O. Symes, W. K. Wills, C. H. Walker and Mr. J. Taylor.

### NINETY-NINTH LIST OF BOOKS.

The figures in brackets refer to the figures after the names of the donors, and show by whom the volumes were presented. The books to which no such figures are attached have either been bought from the Library Fund, or received through the *Journal.*

*Accessory Food Factors, Report on* .. .. .. .. .. .. .. (3) 1919
**Aitchison, R. S.** .. *A Medical Handbook* .. .. .. 5th Ed. 1920
*Albuminuria and War Nephritis among British Troops in France* (3) 1919
*Anærobic Bacteria and Infections, Reports on* .. .. .. .. (3) 1919

**Barbour, D. B. Hart and A. H. F.** *Manual of Gynæcology.* (1) 4th Ed. 1890
**Berkeley and V. Bonney, C.** *Gynæcological Surgery* .. .. 2nd Ed. 1920
**Berkeley and V. Bonney, C.** *Gynæcology in General Practice* [2nd Ed.] 1919
**Cunningham, D. J.** *Manual of Practical Anatomy* (Ed. by A. Robinson) .. .. Vol. I. 7th Ed. 1919
**Cushing, H.** .. .. *Tumours of the Nervus Acusticus* .. .. 1917
*Encyclopædia Medica* .. .. .. .. .. Vol. VI. 2nd Ed. 1919
**Findlay, L.** .. .. *Syphilis in Childhood* .. .. .. .. .. 1919
**Groves, E. W. H.** *Surgical Operations* .. .. .. .. .. .. .. 1919
**Ham, B. B.** .. .. *Sanitary Law* (Ed. by H. R. Kenwood) 8th Ed. 1920
**Harris, W.** .. .. *Electrical Treatment* .. .. .. 3rd Ed. 1919
**Hart, and A. H. F. Barbour, D. B.** *Manual of Gynæcology* (1) 4th Ed. 1890
**Hayes, Marie E.** .. *At Work* .. .. .. .. .. .. .. .. (2) [1909]
**Herman, G. E.** .. *Difficult Labour* (Ed. by C. Oldfield) 6th Ed. 1920
**Horsley and Mary D. Sturge, Sir V.** *Alcohol and the Human Body* 6th Ed. 1920
**Hurst, A. F.** .. *Medical Diseases of the War* .. 2nd Ed. 1918
*Ireland's Crusade against Tuberculosis.* 3 vols. .. .. (2) 1908–9
**Jellett, H.** .. .. *Short Practice of Midwifery* .. (1) 5th Ed. 1908
**Kent and A. J. Rosanoff, G. H.** *A Study of Association in Insanity* (2) 1910
**Laurens, G.** .. .. *Oto-Rhino-Laryngology* (Trans. by H. C. Fox) 1919
**McVail, J. C.** .. *Half a Century of Small Pox and Vaccination* 1919
**Mummery, J. H.** .. *Microscopic Anatomy of the Teeth* .. .. 1919
**Porter, W. G.** .. *Diseases of the Throat, Nose and Ear* (Ed. by A. L. Turner) .. .. .. .. .. .. 3rd Ed. 1919
**Pringle, J. J.** [Ed.] *Atlas of Skin Diseases* .. .. .. .. (1) 1897
**Pye, [W.]** .. .. *Surgical Handicraft* (Ed. by V. Z. Cope) 14th Ed. 1919
**Rosanoff, G. H. Kent and A. J.** *A Study of Association in Insanity* (2) 1910
**Sturge, Sir V. Horsley and Mary D.** *Alcohol and the Human Body* 6th Ed. 1920
**Wallace, J. S.** *Child Welfare* .. .. .. .. .. .. .. .. 1919
**Whitla, Sir W.** .. *A Dictionary of Treatment* .. .. 6th Ed. 1919
**Wilson, R. M. and W. M. T.** *War Diseases and Pensions* .. .. .. 1919
*Wound Shock and Hæmorrhage* .. .. .. .. .. .. .. .. (3) 1919

### TRANSACTIONS, REPORTS, JOURNALS, &c.

American Laryngological Association, Trans. of the .. .. .. .. 1918
American Surgical Association, Trans. of the .. .. Vol. XXXVI. 1918
British Medical Journal, The .. .. .. .. .. .. Vol. I. for 1919
Dermatological Society of London, Trans. of the .. .. .. 1919–20
English Catalogue of Books for 1918 .. .. .. .. .. .. .. .. 1919
Johns Hopkins' Hospital Reports .. .. .. .. .. Vol. XVIII. 1919
Lancet, The .. .. .. .. .. .. .. .. .. .. .. Vol. I. for 1919
Medical Society's Transactions .. .. .. .. .. .. Vol. XLII. 1919
Ophthalmological Transactions .. .. .. .. .. Vol. XXXIX. 1919

### Local Medical Notes.

Examination Successes.—Students of the University have recently passed the following examinations :—

University of Bristol.—*M.B., Ch.B.*—1*st Examination :* L. Dunn, E. F. King, G. M. Minifie, C. P. Porter, G. A. S. Thomas. 2*nd Examination, Part I. :* A. T. Binnie, S. H. Blacker, A. P. Bodman, E. G. Bradbeer, E. R. Clutterbuck, B. A. Crook, B. V. Dawkins, H. M. Dixon, R. H. Dummett, G. R. Dunn, F. R. Edbrooke, N. J. England, P. G. Evans, H. M. Golding, W. A. Gornall, J. L. Griffin, R. C. Hatcher, A. G. Heron, E. G. Hill, J. A. James, F. G. Jenkins, D. E. Joscelyne, P. C. Joscelyne, E. F. King, P. H. Knowles, A. H. Marshall, J. R. N. Lailey, K. F. Platt, A. S. Prowse, A. Skardon, R. A. Sammons, H. L. Shepherd, H. E. Sherwell, H. J. Spreadbury, H. Taylor, R. E. Whitby. *Part II. :* F. H. Bodman, J. M. Evans, J. E. S. Hamilton, F. J. Hector, M. G. Hughes, J. M. Jones, A. J. Keevil, E. C. Kenderdine, F. Langford, C. H. Osmond, D. M. Pullen, D. Staley, I. Williams, B. A. Crook, H. L. Shepherd, H. J. Spreadbury. *Anatomy :* G. B. Bush, C. L. Griffiths, G. S. Mundy, M. Taylor. *Final Examination, Part II. :* G. A. Astley-Weston, H. M. Brown, D. G. Cossham, S. Datta, F. V. Jacques. *Part I. :* K. W. Chan, M. Wadsworth.

D.P.H.—W. D. Scott. *Part I.:* S. H. Kingston, A. D. Symons, G. C. Williams.

Diploma in Dental Surgery.—*2nd Examination:* N. H. Bodenham, K. G. Hyland, W. Bayly, L. C. Bodey, E. E. Lewis. *Part I.:* J. C. L. Phillips.

B.D.S.—*2nd Examination:* G. N. Season.

Conjoint Examining Board.—*Surgery:* F. V. Jacques. *Midwifery:* M. A. Distaso.

L.S.A.—*Medicine, Part II.:* W. E. Neale.

L.D.S., R.C.S. Eng.—*Mechanical Dentistry:* L. H. Buchan, I. Jacobs, E. R. Pfaff, A. J. Constance. *Dental Metallurgy:* L. H. Buchan, C. E. Down, R. B. Hylton, J. C. L. Phillips, H. H. Watkins. *Final Examination, Part I.:* H. F. Buchan, B. Leathlean, E. F. Vowles, I. Jacobs, L. H. Buchan, E. R. Pfaff. *Surgery only:* H. B. Long. *Part II.:* E. E. Wookey, H. B. Long.

Royal College of Physicians of London.—R. C. Clarke, M.B., Ch.B., admitted to the membership.

---

Illustrations.—*Contributors are asked to note that sketches for reproduction as line blocks should be drawn in black Indian ink on white paper, and of the actual size (quarter, half, or full page) they are to be reproduced.*

